# Conscious recall of different aspects of skill memory

**DOI:** 10.3389/fnbeh.2014.00233

**Published:** 2014-07-01

**Authors:** Sunbin Song, Leonardo G. Cohen

**Affiliations:** Human Cortical Physiology and Neurorehabilitation Section, National Institute of Neurological Disorders and Stroke, National Institutes of HealthBethesda, MD, USA

**Keywords:** conscious recall, transition, ordinal, skill learning, implicit, process dissociation procedure, serial reaction time task, sequence learning

## Abstract

Different mechanisms are involved in the formation of memories necessary for daily living. For example, different memory representations are formed for the practiced transitions between key-presses (i.e., pressing key “2” after “3” in “4-3-2-1”) and for the ordinal position of each key-press (i.e., pressing key “2” in the third ordinal position in “4-3-2-1”) in a motor sequence. Whether the resulting transition-based and ordinal-based memories (Song and Cohen, 2014) can be consciously recalled is unknown. Here, we studied subjects who over a week of training and testing formed transition and ordinal-based memory representations of skill for a 12-item sequence of key-presses. Afterwards, subjects were first asked to recall and type the trained sequence and then to perform random key-presses avoiding the trained sequence. The difference in the ability to purposefully recall and avoid a trained sequence represents conscious recall (Destrebecqz and Cleeremans, 2001). We report that (a) the difference in the ability to purposefully recall and to avoid the trained sequence correlated with ordinal-based but not with transition-based memory; (b) subjects with no ability to recall or avoid the trained sequence formed transition-based but not ordinal-based memories; and (c) subjects with full ability to recall and avoid the trained sequence formed both transition-based and ordinal-based memories. We conclude that ordinal-based memory can be voluntarily recalled when transition-based memory cannot, documenting a differential capacity to recall memories forming a motor skill. Understanding that different memories form a motor skill, with different neural substrates (Cohen and Squire, 1980), may help develop novel training strategies in neurorehabilitation of patients with brain lesions.

## Introduction

In our daily lives, we rely on skills that engage motor sequencing such as speaking and driving (Lashley, 1951; Terrace and McGoningle, 1994; Conway and Christiansen, 2001). Humans practicing a sequential motor skill form different memory representations based on the practiced transitions between key-presses (i.e., pressing key “2” after “3” in “4-3-2-1”) and on the ordinal position of each key-press (i.e., pressing key “2” in the third ordinal position in “4-3-2-1”) (Terrace and McGoningle, 1994; Conway and Christiansen, 2001). The evolutionary development (Terrace and McGoningle, 1994) and timeline of formation of these different memory types differ (Song and Cohen, 2014) suggesting differing neural underpinnings. Currently, there is a gap in knowledge on the extent to which transition-based and ordinal-based memories can be consciously recalled. Understanding this issue is important to explore their neural underpinnings and functions.

Here, we studied healthy subjects who, over a week of training and testing formed transition and ordinal-based memory representations of skill for a 12-item sequence of key-presses (Song and Cohen, 2014). Subjects were then tested using the process dissociation procedure (PDP), which measures motoric free recall and suppression to characterize conscious recall (Jacoby, 1998; Destrebecqz and Cleeremans, 2001). Specifically, subjects are instructed to type repeatedly the trained sequence (Inclusion condition) and then to type key-presses randomly specifically avoiding the trained sequence (Exclusion condition). The difference in the ability to purposefully recall (Inclusion) and avoid (Exclusion) the trained sequence defines conscious recall (Destrebecqz and Cleeremans, 2001). This form of skill training followed by motoric recall has proved amenable to the evaluation of conscious recall of different memory types, which was the focus of this study.

## Materials and methods

### Participants

Eighty subjects gave their informed consent to the experimental procedure, which was approved by the National Institute of Neurological Disorders and Stroke (NINDS) Institutional Review Board with minor deviations on testing times being reported to the Internal Review Board (IRB). One subject was excluded due to an error in data collection (total *n* = 79). All subjects were right handed and naïve to the task and had a normal neurological examination as assessed by a credentialed practitioner.

### Design and procedure

#### Formation of the motor skill memory

Subjects were trained on a 12-item sequence using the serial reaction time tasks or SRTT (Goedert and Willingham, 2002; Keele et al., 2003). In this task, subjects press a key with one of four fingers of the right hand in response to a visuo-spatial target appearing in one of four locations on a computer screen. Targets follow a repeating (8 times) 12-item sequence during Pattern blocks and a pseudo-randomized order in Random blocks. Sequences used in Pattern blocks were chosen from a corpus of 563 different possible options (every 12-item sequence in this corpus contained no repetitions and had every key-press represented exactly three times) (Goedert and Willingham, 2002) and those in Random blocks from other members of the corpus chosen at random. Subjects’ motor skill memory tested on the Pattern blocks 1 week after training was related to conscious recall (see below). By 1 week, subjects had completed 10 Pattern blocks in total. Relationship between skill at different time points and sleep was reported before (Song and Cohen, 2014).

Prior to training all subjects were instructed to respond as fast as possible with perfect accuracy and were split in two groups according to the Instructions provided (Intentional and Unintentional groups). Subjects in the Intentional group (*n* = 39, 15 females, 27 +/− 5yo) were informed about the presence of a 12-item sequence, they were asked to memorize. Subjects in the Unintentional group (*n* = 40, 16 females, 28 +/− 7yo) were not. Following each Pattern block, subjects in the Intentional group were asked to reproduce the sequence verbally. At the end of the 1 week test, both Intentional and Unintentional groups were informed about the existence of the 12-item repeating sequence.

Evaluation of transition-based memory involved first assessing Random blocks which do not contain ordinal information but include by chance high frequency transitions present in the trained sequence. As a consequence, certain transitions in Random blocks become higher frequency and others lower frequency, providing a large degree of variance in conditional probabilities of transitions. In each subject, principle component regression that related frequencies of transitioning up to seven items prior (1st–6th order conditional (OC) probabilities (Remillard, 2010)) with response times in Random blocks, was used to construct a model describing the relationship between variance in speed and variance in conditional probabilities of transitions. This model was then applied to estimate transition-based memory in the Pattern block. Ordinal-based memory was assessed by comparing response times on items containing identical transition information across block types. To do this, we would ideally want to identify identical trains seven items in length under the rationale that key-press “3” in “123214-3” in a Pattern block shares the same transition information as a “3” in “123214-3” in a Random block but differs due to the set ordinal position in the Pattern block. Practically, long identical trains across block types were too sparse to directly compare. For this reason, we focused on triplets and quadruplets that were identical between Pattern and Random blocks, and then removed the contributions from higher order transition differences (3rd and higher OC for triplets, 4th and higher OC for quadruplets) (Song and Cohen, 2014).

#### Process dissociation procedure (PDP)

Subjects were instructed to type repeatedly the trained sequence (Inclusion condition) and then to type key-presses randomly specifically avoiding the trained sequence and key-press repetitions (Exclusion condition) in two blocks of 96 key-presses each. The difference in the ability to purposefully recall (Inclusion) and avoid (Exclusion) the trained sequence defined conscious recall (Destrebecqz and Cleeremans, 2001).

##### Standard analysis

Data recorded during the Inclusion and Exclusion conditions was parsed into overlapping bins of 3 key-presses (triplets, bin size = 3). Thus, the 96 key-presses in each condition were composed of 94 overlapping triplets. Frequency of correct generation in a condition was the percentage of the 94 triplets that matched any of the 12 triplets composing the trained sequence (Destrebecqz and Cleeremans, 2001).

##### Generation curves

**Figure 1:**
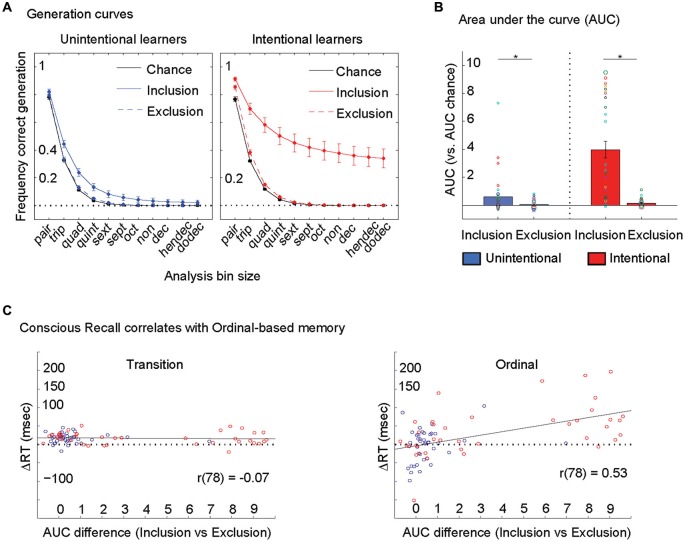
**Conscious recall correlates with ordinal-based memory. (A)** Standard analysis on the PDP focuses on one bin size only (= 3, triplets). Here, we looked at all bin sizes (pairs to dodecs, bin size = 2 through 12) to construct generation curves for each person during Inclusion and Exclusion conditions. In other words, 96 trials generated in each condition was parsed into 95 pairs, 94 triplets, 93 quads, 92 quints and so on and so forth until dodecs (85 dodecs). Within each bin size we counted the frequency of correct generation (i.e., 47 correct/94 triplets = 50%; etc.). At lower bin sizes, frequency of correct generation by chance is high (i.e., chance is 12/36 triplets = 33%) but at higher bin sizes, the chance of correct generation is lower (12/108 quads = 11%, 12/324 quints = 4%, 12/972 sexts = 1%, etc.). Hence the chance generation curve (in black) demonstrates that the effects of chance correct generation at a given bin size decreases progressively as bin size increases from 2 to 12. Generation curves from Inclusion and Exclusion in Unintentional and Intentional learners are plotted here. Error bars are s.e.m. **(B)** Area under the curve (AUC) was measured across generation curves in the Inclusion and Exclusion conditions for each person (individuals plotted here with circles, with larger circles representing more than one subject for that value). At the group level, both Unintentional (in blue) and Intentional (in red) learners showed larger AUCs in Inclusion compared to Exclusion conditions. Error bars are s.e.m.** (C)** The difference in the ability to purposefully recall (Inclusion AUC) and avoid (Exclusion AUC) the trained sequence, that defined conscious recall correlated with the magnitude of ordinal-based memory only (right). Unintentional subjects are represented by blue circles and Intentional subjects by red circles.

We further stratified the PDP data into overlapping bins of 2 through 12 key-presses (pairs to dodecs, bin size = 2 through 12) and evaluated the frequency of correct generation of all bin sizes. At lower bin sizes, frequency of correct generation by chance is high but at higher bin sizes, the chance of correct generation is lower (Figure 1A). By looking at all bin sizes we constructed a generation curve that provided more detailed information on accuracy than in previous work that focused solely on triplets (bin size = 3) (Destrebecqz and Cleeremans, 2001; Wilkinson and Shanks, 2004). Area under the curve (AUC) was measured across generation curves in the Inclusion and Exclusion conditions (Figure 1).

##### Computer modeling

Next, we built computer simulations of generation curves that assumed correct typing of the first 2 items in each sequence, then first 3, 4, 5… up to 12 items in each sequence. As a result, we ended up with 11 simulations of generation curves per person (each person practiced a different sequence from the corpus) that were averaged across all subjects (11 group simulations). Subjects’ AUC for the real generation curves were related to the average ones to estimate the # of items they could correctly recall.

### Statistical analyses

Mauchly’s test was used to test sphericity. Next, data was analyzed using 2 × 2 ANOVA_MixedDesign_ with within subject factors of Condition (Inclusion vs. Exclusion) and between subject factors of Instruction (Intentional vs. Unintentional). *Post-hoc* tests were used after a significant main effect or interaction in the ANOVA. If the sphericity test failed, we applied a Greenhouse-Geisser correction, which is reflected in the main text as a correction to the degrees of freedom. For correlations, Pearson’s *R* is reported. In figures, data is shown as group means + SE and results were considered significant at *p* < 0.05. All statistical analyses were performed in SPSS (SPSS Inc., Chicago IL).

## Results

ANOVA_MD_ revealed a significant effect of Instruction: *F*_(1,77)_ = 29.5, *p* < 0.0001, Condition: *F*_(1,77)_ = 65.6, *p* < 0.0001, and their interaction: *F*_(1,77)_ = 14.8, *p* < 0.0001 on the frequency of correct generation of triplets (Figure 1A, bin size = 3, Standard Analysis). *Post-hoc* testing showed that the frequency of correct generation of triplets in the Inclusion were larger than in the Exclusion condition in both Unintentional (*t*_(39)_ = 3.9, *p* < 0.0001) and Intentional (*t*_(38)_ = 7.0, *p* < 0.0001) groups. Thus, both groups show significant conscious recall.

In relation to the Generation Curves, ANOVA_MD_ revealed a significant effect of Instruction: *F*_(1,77)_ = 28.4, *p* < 0.0001, Condition: *F*_(1,77)_ = 46.3, *p* < 0.0001, and their interaction: *F*_(1,77)_ = 25.8, *p* < 0.0001 on AUCs (Figures 1A,B). *Post-hoc* testing showed that AUCs in the Inclusion were larger than in the Exclusion condition in both Unintentional (*t*_(39)_ = 2.8, *p* < 0.008) and Intentional (*t*_(38)_ = 6.2, *p* < 0.0001) groups. The difference in the ability to purposefully recall (Inclusion) and avoid (Exclusion) the trained sequence, that defined conscious recall correlated with the magnitude of ordinal-based memory only (*r*_(78)_ = 0.53, *p* < 0.0001, Figure 1C). This analysis indicates that conscious recall correlates with ordinal-based but not transition-based memory, an effect more prominent in the Intentional group (Figure 1C).

**Figure 2:**
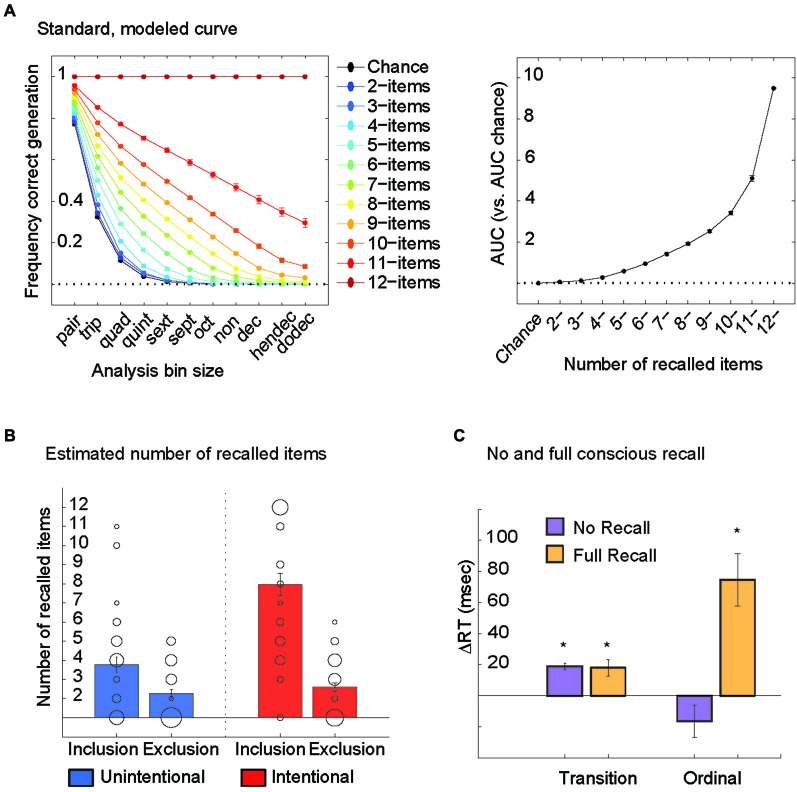
**(A)** Computer simulations were used to construct generation curves that assumed correct typing of the first 2 items in each sequence, then first 3, 4, 5… up to 12 items in each sequence. As a result, we ended up with 11 simulations of generation curves per person (each person practiced a different sequence from the corpus) that were averaged across all subjects (11 group simulations, left). Average AUC values for these 11 group simulations are shown (right). Note that the relationship between generation frequencies and number of items recalled is not linear but exponential. This means that even when the number of items recalled is normally distributed within a group of subjects, an analysis of generation frequencies will skew heavily towards the right. **(B)** Subjects’ actual AUC scores on the PDP were compared to the standard, modeled AUC curve to estimate the number of items they could correctly recall (individuals plotted here with circles, with larger circles representing more than one subject for that value). Subjects from both Unintentional and Intentional groups were stratified into those who could recall all 12 items in the Inclusion and avoid them in the Exclusion condition (full recall) and those who could not recall more items in the Inclusion than in the Exclusion condition (no recall). **(C)** Both no recall and full recall groups showed transition-based memory, and only subjects with full recall showed ordinal-based memory.

Subjects’ AUC scores were compared to a standard, modeled AUC curve to estimate the number of items they could correctly recall (Figures 2A,B). Unintentional learners recalled 4 +/− 3SD (range 0–11) items in the Inclusion and 2 +/− 2SD (range 0–5) items in the Exclusion condition. Intentional learners recalled 8 +/− 4 (range 0–12) items in the Inclusion and 2 +/− 2SD (range 0–6) items in the Exclusion condition (Figure 2B). Recall in the Inclusion but not Exclusion condition correlated with the subjects’ ability to reproduce the sequence verbally following each Pattern block in the Intentional group (*r*_(38)_ = 0.76, *p* < 0.0001).

To further characterize the influence of conscious recall on memory types, we stratified subjects from both Unintentional and Intentional groups into those who could recall all 12 items in the Inclusion and avoid them in the Exclusion condition (full recall) and those who could not recall more items in the Inclusion than in the Exclusion condition (no recall). Both groups showed transition-based memory (one-sample *t*-test, No: *t*_(24)_ = 8.67, *p* < 0.0001; Full: *t*_(12)_ = 3.28, *p* < 0.007) but only subjects with full recall showed ordinal-based memory: *t*_(12)_ = 3.27, *p* < 0.001 (Figure 2C). Hence, no conscious recall was accompanied by only transition-based skill, while full conscious recall was accompanied by both transition-based and ordinal-based skill.

## Discussion

Here, we investigated conscious recall of different aspects of skill memory: specifically, recall of transition-based and ordinal-based representations of skill as measured when learning a sequence of key-presses (Song and Cohen, 2014). Our overall results identified a differential ability to recall these different aspects of skill memory in healthy humans.

First, we found that, consistent with previous literature (Wilkinson and Shanks, 2004), both instructional groups (Intentional and Unintentional) showed significant conscious recall and greater conscious recall in Intentional compared to Unintentional learners using a standard analysis (Figure 1A). A substantial limitation of this approach has been the inability to quantify conscious recall at an individual level. Here, we addressed this issue by introducing a new analysis method that evaluated generation at all bin sizes instead of solely triplets. One substantial advantage of this new approach is that, as bin size increases beyond the standard = 3, the effects of chance correct generation at a given bin size decreases progressively as bin size increases up to 12 (Figure 2A). Though on the whole, we again found significant conscious recall in both groups and greater conscious recall in Intentional compared to Unintentional learners, within each group there was a high degree of inter-individual variance (Figure 2B). This both highlights the close connection between the conscious processes of intention and recall (Frensch, 1998) while also demonstrating their dissociation in that intention to learn did not guarantee a given level of conscious recall. We found that conscious recall correlated with ordinal-based but not transition-based memory (*r*_(78)_ = 0.53 and *r*_(78)_ = −0.07 respectively) and that the correlation of conscious recall with ordinal-based memory was significantly larger than that with transition-based memory (Steiger’s *Z*_H(76)_ = 4.1, *p* < 0.001). Thus, the strong link between conscious recall and ordinal-based memory is likely to explain previously reported relationships with total skill (which includes both aspects of skill memory) (Perruchet and Amorim, 1992).

A limitation of analysis of generation frequencies only is that it does not translate into the actual behavior in this task: the number of items within the trained sequence that each subject can recall. To address this problem, we built computer simulations of generation curves that assumed correct typing of the first 2 items in each sequence, then first 3, 4, 5… up to 12 items in each sequence. The 11 simulations of generation curves per person (each person practiced a different sequence from the corpus) allowed us to characterize the number of items within the trained sequence that each subject could recall (Figure 2A). We found that both full recall and no recall groups showed transition-based memory but only subjects with full recall showed ordinal-based memory. These results indicate that transition-based memory does not support conscious recall.

Our core finding of different conscious recall for different aspects of skill memory is consistent with the concept that transition-based and ordinal-based aspects of skill memory have different neural underpinnings. Evidence for this contention comes from the findings of dissociations in evolutionary development (Terrace and McGoningle, 1994; Conway and Christiansen, 2001) and timeline of formation of transition-based and ordinal-based aspects of skill (Song and Cohen, 2014). Given the contribution of the medial temporal lobe function to consciously recalled memory (Cohen and Squire, 1980), this region is likely influential on ordinal-based memory, an issue for future investigation. In addition to these mechanistic implications, these results raise the hypothesis of selective abnormalities in skill memory learning during neurorehabilitation following brain lesions like stroke or traumatic brain injury. It remains to be determined how neurorehabilitation interventions proposed to improve learning such as interleaved practice schedules (Hanlon, 1996) and non-invasive brain stimulation (Hummel and Cohen, 2006) affect different aspects of procedural memories: transition and ordinal-based. We hypothesize that different interventions could have differential effects on transition vs. ordinal-based aspects of skill. Further research into this topic as well as on the influence of different types of brain lesions on each memory type would be of clinical relevance.

## Conflict of interest statement

The authors declare that the research was conducted in the absence of any commercial or financial relationships that could be construed as a potential conflict of interest.
